# 5-Cyclo­hexyl-2-methyl-3-(3-methyl­phenyl­sulfin­yl)-1-benzo­furan

**DOI:** 10.1107/S1600536814011192

**Published:** 2014-05-21

**Authors:** Hong Dae Choi, Uk Lee

**Affiliations:** a Department of Chemistry, Dongeui University, San 24 Kaya-dong, Busanjin-gu, Busan 614-714, Republic of Korea; b Department of Chemistry, Pukyong National University, 599-1 Daeyeon 3-dong, Nam-gu, Busan 608-737, Republic of Korea

## Abstract

In the title compound, C_22_H_24_O_2_S, the cyclo­hexyl ring adopts a chair conformation. The dihedral angle between the mean planes of the benzo­furan and 3-methyl­phenyl moieties is 86.48 (4)°. In the crystal, mol­ecules are connected along the *a*-axis direction by two different inversion-generated pairs of C—H⋯π and C—H⋯O inter­actions.

## Related literature   

For background information and the crystal structures of related compounds, see: Choi *et al.* (2012 ▶, 2013 ▶, 2014 ▶).
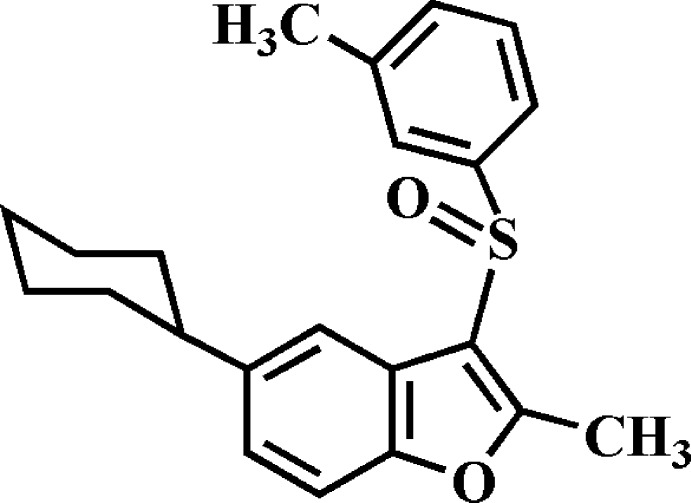


## Experimental   

### 

#### Crystal data  

C_22_H_24_O_2_S
*M* *_r_* = 352.47
Triclinic, 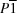

*a* = 8.8562 (2) Å
*b* = 10.3095 (2) Å
*c* = 11.1248 (2) Å
α = 91.147 (1)°
β = 113.425 (1)°
γ = 98.036 (1)°
*V* = 919.66 (3) Å^3^
*Z* = 2
Mo *K*α radiation
μ = 0.19 mm^−1^
*T* = 173 K
0.40 × 0.39 × 0.32 mm

#### Data collection  

Bruker SMART APEXII CCD diffractometer
Absorption correction: multi-scan (*SADABS*; Bruker, 2009 ▶) *T*_min_ = 0.685, *T*_max_ = 0.746
16991 measured reflections
4585 independent reflections
3954 reflections with *I* > 2σ(*I*)
*R*_int_ = 0.026

#### Refinement  

*R*[*F* ^2^ > 2σ(*F* ^2^)] = 0.043
*wR*(*F* ^2^) = 0.113
*S* = 1.03
4585 reflections
228 parameters
H-atom parameters constrained
Δρ_max_ = 0.36 e Å^−3^
Δρ_min_ = −0.29 e Å^−3^

### 

Data collection: *APEX2* (Bruker, 2009 ▶); cell refinement: *SAINT* (Bruker, 2009 ▶); data reduction: *SAINT*; program(s) used to solve structure: *SHELXS97* (Sheldrick, 2008 ▶); program(s) used to refine structure: *SHELXL97* (Sheldrick, 2008 ▶); molecular graphics: *ORTEP-3 for Windows* (Farrugia, 2012 ▶) and *DIAMOND* (Brandenburg, 1998 ▶); software used to prepare material for publication: *SHELXL97*.

## Supplementary Material

Crystal structure: contains datablock(s) I. DOI: 10.1107/S1600536814011192/ld2128sup1.cif

Structure factors: contains datablock(s) I. DOI: 10.1107/S1600536814011192/ld2128Isup2.hkl

Supporting information file. DOI: 10.1107/S1600536814011192/ld2128Isup3.cml

CCDC reference: 1003285

Additional supporting information:  crystallographic information; 3D view; checkCIF report

## Figures and Tables

**Table 1:** Hydrogen-bond geometry (Å, °) *Cg*1 is the centroid of the C2–C7 benzene ring.

*D*—H⋯*A*	*D*—H	H⋯*A*	*D*⋯*A*	*D*—H⋯*A*
C22—H22*B*⋯O2^i^	0.98	2.54	3.295 (2)	134
C14—H14*A*⋯*Cg*1^ii^	0.99	2.91	3.607 (2)	128

Symmetry codes: (i) 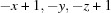; (ii) 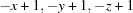.

**Fig. 1.**
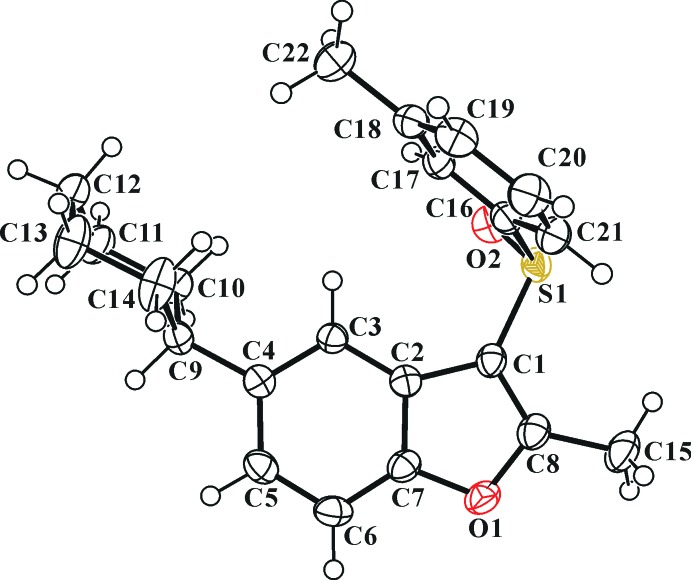
The molecular structure of the title molecule with the atom numbering scheme The displacement ellipsoids are drawn at the 50% probability level. The hydrogen atoms are presented as small spheres of arbitrary radius.

**Fig. 2.**
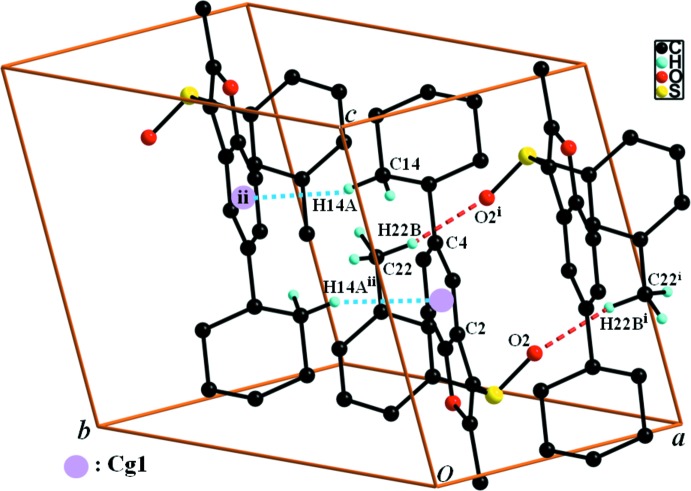
A view of the C—H···O and C—H···π interactions (dotted lines) in the crystal structure of the title compound. H atoms non-participating in hydrogen-bonding were omitted for clarity. [Symmetry codes: (i) - x + 1, - y, - z + 1; (ii) - x + 1,- y + 1,- z + 1.]

## supplementary crystallographic information

### 1. Comment

As a part of our continuing study of 5-cyclohexyl-2-methyl-1-benzofuran derivatives containing 4-methylphenylsulfinyl (Choi *et al.*, 2012), 3-fluorophenylsulfinyl (Choi *et al.*, 2013) and 3-bromophenylsulfinyl (Choi *et al.*, 2014) substituents in 3-position, we report here on the crystal structure of the title compound.

In the title molecule (Fig. 1), the benzofuran unit is essentially planar, with a mean deviation of 0.005 (1) Å from the least-squares plane defined by the nine constituent atoms. The 3-methylphenyl ring is essentially planar, with a mean deviation of 0.006 (1) Å from the least-squares plane defined by the six constituent atoms. The cyclohexyl ring is in the chair form. The dihedral angle formed by the benzofuran ring system and the 3-methylphenyl ring is 86.48 (4)°. In the crystal structure (Fig. 2), molecules are connected *via* two different inversion-generated pairs of C—H···π and C—H···O interactions (Table 1, Cg1 is the centroid of the C2–C7 benzene ring), forming supramolecular stacks running along the a-axis direction.

### 2. Experimental

3-Chloroperoxybenzoic acid (77%, 224 mg, 1.0 mmol) was added in small portions to a stirred solution of 5-cyclohexyl-2-methyl-3-(3-methylphenylsulfanyl)-1-benzofuran (302 mg, 0.9 mmol) in dichloromethane (30 mL) at 273 K. After being stirred at room temperature for 4h, the mixture was washed with saturated sodium bicarbonate solution and the organic layer was separated, dried over magnesium sulfate, filtered and concentrated at reduced pressure. The residue was purified by column chromatography (hexane–ethyl acetate, 2:1 *v*/*v*) to afford the title compound as a colorless solid [yield 75%, m.p. 390–391 K; *R*_f_ = 0.56 (hexane-ethyl acetate, 2:1 *v*/*v*)]. Single crystals suitable for X-ray diffraction were prepared by slow evaporation of a solution of the title compound in acetone at room temperature.

### 3. Refinement

All H atoms were positioned geometrically and refined using a riding model, with C—H = 0.95 Å for aryl, 1.00 Å for methine, 0.99 Å for methylene and 0.98 Å for methyl H atoms, respectively. *U*_iso_ (H) = 1.2*U*_eq_ (C) for aryl, methine and methylene, and 1.5*U*_eq_ (C) for methyl H atoms. The positions of methyl hydrogens were optimized using the SHELXL-97's command AFIX 137 (Sheldrick, 2008).

### Crystal data

**Table:**

C_22_H_24_O_2_S	*Z* = 2
*M**_r_* = 352.47	*F*(000) = 376
Triclinic, *P*1	*D*_x_ = 1.273 Mg m^−^^3^
Hall symbol: -P 1	Melting point = 391–390 K
*a* = 8.8562 (2) Å	Mo *K*α radiation, λ = 0.71073 Å
*b* = 10.3095 (2) Å	Cell parameters from 6026 reflections
*c* = 11.1248 (2) Å	θ = 2.5–28.3°
α = 91.147 (1)°	µ = 0.19 mm^−^^1^
β = 113.425 (1)°	*T* = 173 K
γ = 98.036 (1)°	Block, colourless
*V* = 919.66 (3) Å^3^	0.40 × 0.39 × 0.32 mm

### Data collection

**Table:**

Bruker SMART APEXII CCD diffractometer	4585 independent reflections
Radiation source: rotating anode	3954 reflections with *I* > 2σ(*I*)
Graphite multilayer monochromator	*R*_int_ = 0.026
Detector resolution: 10.0 pixels mm^-1^	θ_max_ = 28.4°, θ_min_ = 2.0°
φ and ω scans	*h* = −11→11
Absorption correction: multi-scan (*SADABS*; Bruker, 2009)	*k* = −13→13
*T*_min_ = 0.685, *T*_max_ = 0.746	*l* = −14→13
16991 measured reflections	

### Refinement

**Table:**

Refinement on *F*^2^	Primary atom site location: structure-invariant direct methods
Least-squares matrix: full	Secondary atom site location: difference Fourier map
*R*[*F*^2^ > 2σ(*F*^2^)] = 0.043	Hydrogen site location: difference Fourier map
*wR*(*F*^2^) = 0.113	H-atom parameters constrained
*S* = 1.03	*w* = 1/[σ^2^(*F*_o_^2^) + (0.0525*P*)^2^ + 0.3576*P*] where *P* = (*F*_o_^2^ + 2*F*_c_^2^)/3
4585 reflections	(Δ/σ)_max_ < 0.001
228 parameters	Δρ_max_ = 0.36 e Å^−^^3^
0 restraints	Δρ_min_ = −0.29 e Å^−^^3^

### Special details

**Table:**

Geometry. All esds (except the esd in the dihedral angle between two l.s. planes) are estimated using the full covariance matrix. The cell esds are taken into account individually in the estimation of esds in distances, angles and torsion angles; correlations between esds in cell parameters are only used when they are defined by crystal symmetry. An approximate (isotropic) treatment of cell esds is used for estimating esds involving l.s. planes.
Refinement. Refinement of F^2^ against ALL reflections. The weighted R-factor wR and goodness of fit S are based on F^2^, conventional R-factors R are based on F, with F set to zero for negative F^2^. The threshold expression of F^2^ > 2sigma(F^2^) is used only for calculating R-factors(gt) etc. and is not relevant to the choice of reflections for refinement. R-factors based on F^2^ are statistically about twice as large as those based on F, and R- factors based on ALL data will be even larger.

### Fractional atomic coordinates and isotropic or equivalent isotropic displacement parameters (Å^2^)

**Table:**

	*x*	*y*	*z*	*U*_iso_*/*U*_eq_	
S1	0.33525 (4)	0.01712 (3)	0.19341 (3)	0.02995 (11)	
O1	0.54828 (13)	0.32465 (11)	0.08241 (10)	0.0345 (2)	
O2	0.45096 (14)	−0.04957 (11)	0.30068 (11)	0.0386 (3)	
C1	0.44786 (17)	0.16556 (14)	0.17900 (13)	0.0282 (3)	
C2	0.56092 (16)	0.26407 (14)	0.28182 (13)	0.0263 (3)	
C3	0.61829 (17)	0.28088 (14)	0.41837 (13)	0.0263 (3)	
H3	0.5820	0.2165	0.4651	0.032*	
C4	0.72974 (17)	0.39386 (14)	0.48490 (13)	0.0271 (3)	
C5	0.78181 (18)	0.48692 (15)	0.41363 (15)	0.0323 (3)	
H5	0.8576	0.5637	0.4601	0.039*	
C6	0.72745 (19)	0.47179 (16)	0.27813 (15)	0.0342 (3)	
H6	0.7640	0.5356	0.2309	0.041*	
C7	0.61774 (18)	0.35936 (15)	0.21611 (14)	0.0292 (3)	
C8	0.44668 (19)	0.20622 (16)	0.06342 (14)	0.0322 (3)	
C9	0.79399 (18)	0.41763 (14)	0.63272 (14)	0.0285 (3)	
H9	0.8804	0.4985	0.6605	0.034*	
C10	0.8773 (2)	0.30491 (15)	0.70293 (14)	0.0328 (3)	
H10A	0.7954	0.2227	0.6746	0.039*	
H10B	0.9700	0.2930	0.6775	0.039*	
C11	0.9449 (2)	0.33006 (17)	0.85189 (15)	0.0391 (4)	
H11A	0.9915	0.2524	0.8934	0.047*	
H11B	1.0363	0.4062	0.8814	0.047*	
C12	0.8107 (2)	0.35721 (17)	0.89605 (16)	0.0405 (4)	
H12A	0.8607	0.3800	0.9922	0.049*	
H12B	0.7264	0.2770	0.8770	0.049*	
C13	0.7266 (2)	0.4689 (2)	0.82687 (16)	0.0478 (5)	
H13A	0.8081	0.5513	0.8543	0.057*	
H13B	0.6346	0.4806	0.8532	0.057*	
C14	0.6574 (2)	0.4421 (2)	0.67754 (16)	0.0469 (5)	
H14A	0.6076	0.5184	0.6353	0.056*	
H14B	0.5683	0.3644	0.6492	0.056*	
C15	0.3574 (2)	0.15033 (19)	−0.07522 (15)	0.0434 (4)	
H15A	0.3011	0.0608	−0.0778	0.065*	
H15B	0.4375	0.1483	−0.1155	0.065*	
H15C	0.2746	0.2050	−0.1239	0.065*	
C16	0.20315 (17)	0.08466 (13)	0.25708 (14)	0.0271 (3)	
C17	0.23952 (17)	0.08488 (13)	0.38974 (13)	0.0267 (3)	
H17	0.3339	0.0498	0.4467	0.032*	
C18	0.13706 (18)	0.13682 (14)	0.43984 (14)	0.0293 (3)	
C19	−0.00153 (19)	0.18503 (15)	0.35310 (16)	0.0349 (3)	
H19	−0.0723	0.2208	0.3858	0.042*	
C20	−0.03811 (19)	0.18184 (17)	0.21998 (16)	0.0384 (4)	
H20	−0.1339	0.2147	0.1623	0.046*	
C21	0.06364 (19)	0.13129 (15)	0.17043 (15)	0.0341 (3)	
H21	0.0387	0.1285	0.0790	0.041*	
C22	0.1782 (2)	0.14006 (17)	0.58434 (16)	0.0389 (4)	
H22A	0.2439	0.2255	0.6271	0.058*	
H22B	0.2428	0.0701	0.6221	0.058*	
H22C	0.0748	0.1268	0.5983	0.058*	

### Atomic displacement parameters (Å^2^)

**Table:**

	*U*^11^	*U*^22^	*U*^33^	*U*^12^	*U*^13^	*U*^23^
S1	0.03221 (19)	0.02785 (18)	0.02855 (18)	0.00229 (14)	0.01213 (14)	−0.00417 (13)
O1	0.0349 (6)	0.0442 (6)	0.0278 (5)	0.0052 (5)	0.0166 (4)	0.0054 (4)
O2	0.0401 (6)	0.0350 (6)	0.0432 (6)	0.0124 (5)	0.0173 (5)	0.0045 (5)
C1	0.0270 (7)	0.0325 (7)	0.0256 (6)	0.0044 (6)	0.0116 (5)	−0.0018 (5)
C2	0.0231 (6)	0.0286 (7)	0.0288 (7)	0.0053 (5)	0.0120 (5)	0.0013 (5)
C3	0.0256 (6)	0.0271 (7)	0.0265 (6)	0.0036 (5)	0.0111 (5)	0.0024 (5)
C4	0.0238 (6)	0.0272 (7)	0.0292 (7)	0.0063 (5)	0.0088 (5)	0.0027 (5)
C5	0.0259 (7)	0.0298 (7)	0.0367 (8)	0.0012 (6)	0.0091 (6)	0.0033 (6)
C6	0.0302 (7)	0.0355 (8)	0.0388 (8)	0.0025 (6)	0.0165 (6)	0.0102 (6)
C7	0.0270 (7)	0.0362 (8)	0.0269 (7)	0.0065 (6)	0.0132 (5)	0.0042 (6)
C8	0.0308 (7)	0.0399 (8)	0.0281 (7)	0.0066 (6)	0.0141 (6)	−0.0005 (6)
C9	0.0269 (7)	0.0255 (7)	0.0281 (7)	0.0027 (5)	0.0065 (5)	0.0002 (5)
C10	0.0379 (8)	0.0329 (7)	0.0302 (7)	0.0124 (6)	0.0141 (6)	0.0043 (6)
C11	0.0440 (9)	0.0431 (9)	0.0306 (8)	0.0173 (7)	0.0120 (7)	0.0083 (7)
C12	0.0454 (9)	0.0449 (9)	0.0312 (8)	0.0022 (7)	0.0175 (7)	−0.0031 (7)
C13	0.0471 (10)	0.0627 (12)	0.0334 (8)	0.0247 (9)	0.0115 (7)	−0.0078 (8)
C14	0.0398 (9)	0.0667 (12)	0.0322 (8)	0.0278 (9)	0.0066 (7)	−0.0065 (8)
C15	0.0473 (9)	0.0584 (11)	0.0259 (7)	0.0072 (8)	0.0168 (7)	−0.0021 (7)
C16	0.0263 (7)	0.0242 (6)	0.0287 (7)	0.0003 (5)	0.0102 (5)	−0.0003 (5)
C17	0.0248 (6)	0.0257 (6)	0.0278 (7)	0.0037 (5)	0.0089 (5)	0.0025 (5)
C18	0.0301 (7)	0.0272 (7)	0.0317 (7)	0.0031 (6)	0.0140 (6)	0.0031 (5)
C19	0.0298 (7)	0.0345 (8)	0.0431 (8)	0.0083 (6)	0.0165 (6)	0.0047 (6)
C20	0.0287 (7)	0.0424 (9)	0.0399 (8)	0.0109 (7)	0.0076 (6)	0.0100 (7)
C21	0.0319 (7)	0.0374 (8)	0.0281 (7)	0.0044 (6)	0.0073 (6)	0.0054 (6)
C22	0.0431 (9)	0.0451 (9)	0.0350 (8)	0.0130 (7)	0.0204 (7)	0.0049 (7)

### Geometric parameters (Å, º)

**Table:**

S1—O2	1.4847 (11)	C11—H11B	0.9900
S1—C1	1.7528 (15)	C12—C13	1.513 (2)
S1—C16	1.7950 (14)	C12—H12A	0.9900
O1—C8	1.3698 (19)	C12—H12B	0.9900
O1—C7	1.3815 (17)	C13—C14	1.529 (2)
C1—C8	1.357 (2)	C13—H13A	0.9900
C1—C2	1.4494 (19)	C13—H13B	0.9900
C2—C7	1.391 (2)	C14—H14A	0.9900
C2—C3	1.3953 (19)	C14—H14B	0.9900
C3—C4	1.3935 (19)	C15—H15A	0.9800
C3—H3	0.9500	C15—H15B	0.9800
C4—C5	1.400 (2)	C15—H15C	0.9800
C4—C9	1.5129 (19)	C16—C17	1.3797 (19)
C5—C6	1.386 (2)	C16—C21	1.387 (2)
C5—H5	0.9500	C17—C18	1.395 (2)
C6—C7	1.373 (2)	C17—H17	0.9500
C6—H6	0.9500	C18—C19	1.390 (2)
C8—C15	1.484 (2)	C18—C22	1.499 (2)
C9—C10	1.5241 (19)	C19—C20	1.383 (2)
C9—C14	1.529 (2)	C19—H19	0.9500
C9—H9	1.0000	C20—C21	1.379 (2)
C10—C11	1.524 (2)	C20—H20	0.9500
C10—H10A	0.9900	C21—H21	0.9500
C10—H10B	0.9900	C22—H22A	0.9800
C11—C12	1.511 (2)	C22—H22B	0.9800
C11—H11A	0.9900	C22—H22C	0.9800
			
O2—S1—C1	107.71 (7)	C11—C12—H12A	109.4
O2—S1—C16	107.13 (7)	C13—C12—H12A	109.4
C1—S1—C16	97.97 (6)	C11—C12—H12B	109.4
C8—O1—C7	106.36 (11)	C13—C12—H12B	109.4
C8—C1—C2	107.14 (13)	H12A—C12—H12B	108.0
C8—C1—S1	124.02 (12)	C12—C13—C14	111.37 (14)
C2—C1—S1	128.81 (11)	C12—C13—H13A	109.4
C7—C2—C3	119.30 (13)	C14—C13—H13A	109.4
C7—C2—C1	104.68 (12)	C12—C13—H13B	109.4
C3—C2—C1	136.02 (13)	C14—C13—H13B	109.4
C4—C3—C2	118.83 (13)	H13A—C13—H13B	108.0
C4—C3—H3	120.6	C13—C14—C9	111.25 (13)
C2—C3—H3	120.6	C13—C14—H14A	109.4
C3—C4—C5	119.41 (13)	C9—C14—H14A	109.4
C3—C4—C9	120.91 (13)	C13—C14—H14B	109.4
C5—C4—C9	119.67 (13)	C9—C14—H14B	109.4
C6—C5—C4	122.75 (14)	H14A—C14—H14B	108.0
C6—C5—H5	118.6	C8—C15—H15A	109.5
C4—C5—H5	118.6	C8—C15—H15B	109.5
C7—C6—C5	116.14 (14)	H15A—C15—H15B	109.5
C7—C6—H6	121.9	C8—C15—H15C	109.5
C5—C6—H6	121.9	H15A—C15—H15C	109.5
C6—C7—O1	125.75 (13)	H15B—C15—H15C	109.5
C6—C7—C2	123.57 (13)	C17—C16—C21	121.73 (13)
O1—C7—C2	110.68 (13)	C17—C16—S1	119.36 (10)
C1—C8—O1	111.14 (13)	C21—C16—S1	118.88 (11)
C1—C8—C15	133.36 (16)	C16—C17—C18	119.67 (13)
O1—C8—C15	115.49 (14)	C16—C17—H17	120.2
C4—C9—C10	111.95 (12)	C18—C17—H17	120.2
C4—C9—C14	112.42 (12)	C19—C18—C17	118.50 (13)
C10—C9—C14	109.64 (13)	C19—C18—C22	121.62 (13)
C4—C9—H9	107.5	C17—C18—C22	119.88 (13)
C10—C9—H9	107.5	C20—C19—C18	121.14 (14)
C14—C9—H9	107.5	C20—C19—H19	119.4
C11—C10—C9	111.93 (12)	C18—C19—H19	119.4
C11—C10—H10A	109.2	C21—C20—C19	120.42 (14)
C9—C10—H10A	109.2	C21—C20—H20	119.8
C11—C10—H10B	109.2	C19—C20—H20	119.8
C9—C10—H10B	109.2	C20—C21—C16	118.51 (14)
H10A—C10—H10B	107.9	C20—C21—H21	120.7
C12—C11—C10	111.65 (13)	C16—C21—H21	120.7
C12—C11—H11A	109.3	C18—C22—H22A	109.5
C10—C11—H11A	109.3	C18—C22—H22B	109.5
C12—C11—H11B	109.3	H22A—C22—H22B	109.5
C10—C11—H11B	109.3	C18—C22—H22C	109.5
H11A—C11—H11B	108.0	H22A—C22—H22C	109.5
C11—C12—C13	111.11 (14)	H22B—C22—H22C	109.5
			
O2—S1—C1—C8	131.33 (13)	C7—O1—C8—C15	179.61 (13)
C16—S1—C1—C8	−117.76 (13)	C3—C4—C9—C10	56.24 (17)
O2—S1—C1—C2	−46.28 (14)	C5—C4—C9—C10	−124.23 (14)
C16—S1—C1—C2	64.63 (13)	C3—C4—C9—C14	−67.70 (18)
C8—C1—C2—C7	0.82 (15)	C5—C4—C9—C14	111.83 (16)
S1—C1—C2—C7	178.75 (11)	C4—C9—C10—C11	178.94 (13)
C8—C1—C2—C3	−179.08 (15)	C14—C9—C10—C11	−55.57 (17)
S1—C1—C2—C3	−1.2 (2)	C9—C10—C11—C12	55.45 (19)
C7—C2—C3—C4	0.74 (19)	C10—C11—C12—C13	−54.59 (19)
C1—C2—C3—C4	−179.36 (14)	C11—C12—C13—C14	55.3 (2)
C2—C3—C4—C5	−0.31 (19)	C12—C13—C14—C9	−56.6 (2)
C2—C3—C4—C9	179.22 (12)	C4—C9—C14—C13	−178.73 (14)
C3—C4—C5—C6	−0.2 (2)	C10—C9—C14—C13	56.05 (19)
C9—C4—C5—C6	−179.71 (13)	O2—S1—C16—C17	9.59 (13)
C4—C5—C6—C7	0.2 (2)	C1—S1—C16—C17	−101.79 (12)
C5—C6—C7—O1	−179.97 (13)	O2—S1—C16—C21	−168.25 (12)
C5—C6—C7—C2	0.3 (2)	C1—S1—C16—C21	80.37 (13)
C8—O1—C7—C6	−179.85 (14)	C21—C16—C17—C18	−2.0 (2)
C8—O1—C7—C2	−0.05 (15)	S1—C16—C17—C18	−179.79 (11)
C3—C2—C7—C6	−0.7 (2)	C16—C17—C18—C19	1.1 (2)
C1—C2—C7—C6	179.33 (13)	C16—C17—C18—C22	−178.62 (13)
C3—C2—C7—O1	179.45 (11)	C17—C18—C19—C20	0.1 (2)
C1—C2—C7—O1	−0.47 (15)	C22—C18—C19—C20	179.84 (15)
C2—C1—C8—O1	−0.90 (16)	C18—C19—C20—C21	−0.5 (3)
S1—C1—C8—O1	−178.95 (10)	C19—C20—C21—C16	−0.3 (2)
C2—C1—C8—C15	−179.66 (16)	C17—C16—C21—C20	1.6 (2)
S1—C1—C8—C15	2.3 (3)	S1—C16—C21—C20	179.37 (12)
C7—O1—C8—C1	0.61 (16)		

### Hydrogen-bond geometry (Å, º)

Cg1 is the centroid of the C2–C7 benzene ring.

**Table:**

*D*—H···*A*	*D*—H	H···*A*	*D*···*A*	*D*—H···*A*
C22—H22*B*···O2^i^	0.98	2.54	3.295 (2)	134
C14—H14*A*···*Cg*1^ii^	0.99	2.91	3.607 (2)	128

Symmetry codes: (i) −*x*+1, −*y*, −*z*+1; (ii) −*x*+1, −*y*+1, −*z*+1.
